# Semaglutide Inducing Resolution of Proliferative Diabetic Retinopathy: A Case Report

**DOI:** 10.1155/crop/5834769

**Published:** 2024-12-10

**Authors:** Daniel Cool, James Coventon, Abhishek Sharma

**Affiliations:** ^1^Ophthalmology Department, Princess Alexandra Hospital, Brisbane, Queensland, Australia; ^2^Ophthalmology Department, Cairns Hospital, Cairns, Queensland, Australia; ^3^Queensland Eye Institute, Brisbane, Queensland, Australia

**Keywords:** diabetic macular odema (DMO), diabetic retinopathy, GLP-1 receptor agonists, proliferative diabetic retinopathy (PDR), semaglutide

## Abstract

**Purpose:** To describe a case of regression of proliferative diabetic retinopathy (PDR) following treatment with semaglutide.

**Methods:** Case report.

**Results:** The case describes a 47-year-old woman with Type 2 diabetes, obesity, hypertension, and dyslipidaemia who had difficulty controlling her blood sugar levels despite oral hypoglycaemic medications. She presented with PDR in her right eye and severe nonproliferative diabetic retinopathy (NPDR) in her left eye. After missing her follow-up appointment for panretinal photocoagulation (PRP), her general practitioner initiated semaglutide therapy. Despite minimal changes in glycaemic control, the patient exhibited resolution of neovascularisation in her right eye and improved diabetic macular oedema (DMO) within 6 weeks of semaglutide therapy.

**Conclusion:** This case report suggests a potential independent role for semaglutide in managing PDR.

**Summary**

• This case report demonstrates semaglutide inducing resolution of proliferative diabetic retinopathy (PDR) in a short observation interval.
• This contrasts with previous evidence suggesting GLP-1 agonists may worsen PDR.

## 1. Background

PDR is a complication of diabetes mellitus resulting in the formation of new blood vessels in the retina that can lead to sight-threatening complications such as vitreous haemorrhage and tractional retinal detachment. PDR results from retinal ischaemia and compensatory vascular endothelial growth factor (VEGF) secretion from hypoxic cells, driving the generation of new blood vessels to reperfuse the retina. Traditionally, the definitive treatment for PDR is panretinal photocoagulation (PRP) to necrose ischaemic retinal cells and terminate VEGF production [1], and more recently, Anti-VEGF intravitreal injection therapy has been demonstrated as a safe and effective alternative to PRP [2]. Aside from achieving suitable glycaemic control to prevent onset, there are no reliable systemic medical treatments available for PDR. We present a case of regressed PDR from the effect of semaglutide independent of her glycaemic control. We believe this represents a yet unidentified mechanism of action of semaglutide to induce this regression and treat PDR.

## 2. Case

L.P. is a 47-year-old Papua New Guinean woman who presented to a private retinal clinic with PDR on the background of Type 2 diabetes. She reports difficulty with blood sugar control on oral hypoglycaemics (OHGs) and progressively worsening visual acuity. She has a background of obesity, hypertension, and dyslipidaemia. She has no other known microvascular complications of diabetes. She was treated with OHG's only, taking Xigduo XR (metformin: 1000 mg; dapagliflozin: 10 mg) oculus dexter (OD), gliclazide (30 mg) OD, atorvastatin (40 mg) OD, telmisartan (40 mg)/HCT (12.5 mg), and aspirin (100 mg) daily. At the time of reporting, her weight was 80 kg, and her height was 155 cm, making her BMI 33.3, thus qualifying her with Grade 1 obesity.

On examination, her corrected visual acuity was 6/9-1 OD and 6/7.5-2 oculus sinister (OS). Her intraocular pressure was normotensive 9 and 10 right and left, respectively, and her pupils were noted to be equal and reactive prior to dilation. Anterior segments were phakic, deep, and quiet bilaterally with no evidence of neovascularisation nor cataract. In the right eye, she was found to have PDR with high-risk neovascularisation at the disc. She had a mild diabetic papillitis. In the left eye, there was severe nonproliferative diabetic retinopathy (NPDR) with no neovascularisation. Her optical coherence tomography (OCT) demonstrated bilateral diabetic macular oedema (DMO); however, her visual acuity derangement was insufficient to commence intravitreal injections [3].

L.P. was scheduled for a fundus fluorescein angiogram (FFA) to guide PRP to address her PDR. Unfortunately, she missed her follow-up appointment due to an intercurrent respiratory illness which resolved without intervention. On account of her worsening diabetic complications and obesity, her GP commenced her on weekly semaglutide, and she had received 4 doses prior to her delayed follow-up 6 weeks later.

**Figure 1:**
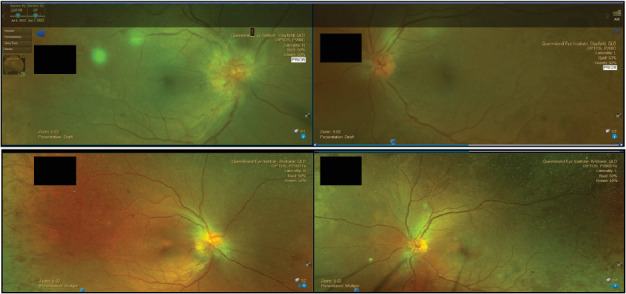
False colour photographs demonstrating regression of neovascularisation. The Top 2 images were taken on 7/6/22 and the bottom on 4/7/22.

Subjectively, she reported minimal change to her home blood glucose levels and a mild amount of weight loss. Despite her weight loss, her HbA1c was largely unaffected by the addition of semaglutide to her regimen, with results both pre (5/5/22) and post (1/8/22) treatment initiation being 9.0, as demonstrated in Table 1. Despite minimal change in these parameters, L.P.'s examination 6 weeks later found regression of her disc neovascularisation (NVD). The pre and postsemaglutide false-colour photographs are visible in Figure 1.

**Figure 2:**
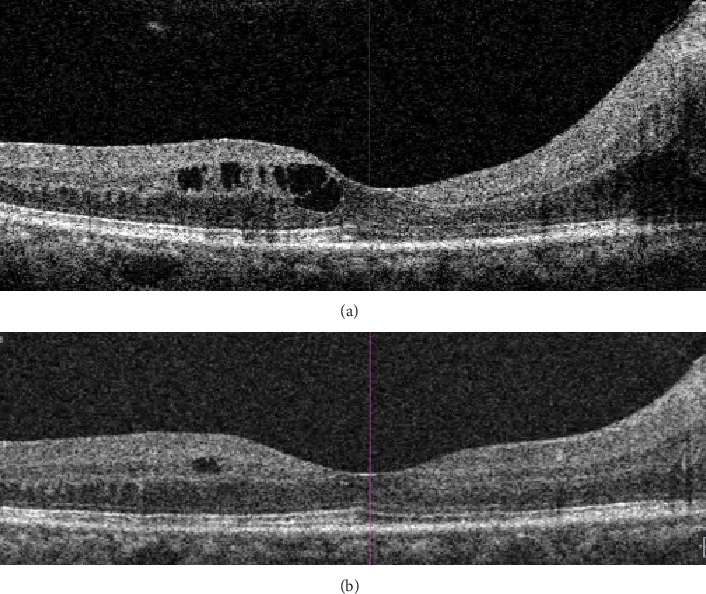
OCT through the left fovea (horizontal slice). (a) 7/6/22 compared to (b) 4/7/22.

At the 6-week review from the initial presentation, her best corrected visual acuity (BCVA) was 6/7.5 in the right eye and 6/6 in the left eye. Her anterior segments remained quiet with no neovascularisation. Her right optic NVD had shown interval reduction, with no observable tractional bands, reduced disc swelling, and nil obvious haemorrhages at the macula, disc, or periphery. There was also an improvement in DMO at this review compared to the previous. This is shown in Figure 2.

Given the marked improvement of proliferative retinopathy in the right eye, as well as improved DMO, the decision was made that PRP or intravitreal therapy was no longer warranted.

## 3. Discussion

This case report demonstrates a previously unreported potential benefit of semaglutide in the regression of PDR [4]. It is in fact contrary to other reports claiming it may in fact worsen diabetic retinopathy.

Semaglutide is a glucagon-like peptide 1 or incretin mimetic that improves glycaemic control and achieves weight loss through a complex mechanism of action [5]. It is delivered via once-weekly dosing, augments insulin secretion, inhibits glucagon secretion, and limits hepatic gluconeogenesis. It has shown enormous efficacy in improving HbA1c measurements and achieving weight loss through the international SUSTAIN trial [6]. Known adverse effects of subcutaneous administration of semaglutide include gastrointestinal disorders most commonly. These include nausea, diarrhoea, and cholelithiasis. More severe complications are rare but include pancreatitis, acute kidney injury, and hypoglycaemia [7].

This case report contradicts some previous ophthalmic evidence of semaglutide. For instance, initiating semaglutide has previously demonstrated an acute worsening of diabetic retinopathy. This is theorised to be mediated by the known phenomenon of intensive glycaemic control improvements acutely worsening diabetic retinopathy in the first 6 months [8], although the exact mechanism of this remains speculative [9]. The SUSTAIN trial demonstrated the progression of diabetic retinopathy in the SUSTAIN 6 data. It is important to note that the inclusion criteria of this group did not preclude patients with pre-existing retinopathy or an upper limit of HbA1c, as was the case in SUSTAIN 1-5 data [2]. This means that the risk of progression to retinopathy in diabetics with current healthy retinas who take semaglutide is unknown.

The potential by which semaglutide might induce regression of PDR may include its known metabolic and vascular effects. As a glucagon-like peptide-1 receptor agonist, it primarily works by enhancing glucose-dependent insulin secretion, inhibiting glucagon release, and slowing gastric emptying, leading to improved glycaemic control [10]. Since hyperglycaemia is a key driver of diabetic retinopathy progression, better glucose regulation could directly reduce the retinal microvascular damage responsible for PDR development. Additionally, glucagon-like peptide-1 receptor agonists are known to have anti-inflammatory and antioxidative properties [11], which may contribute to stabilising or regressing the microvascular complications in PDR by reducing chronic inflammation and oxidative stress.

Furthermore, semaglutide may indirectly influence the levels of VEGF [12]. By improving metabolic parameters, semaglutide could reduce the production of VEGF in retinal tissues, thus limiting pathological neovascularisation. There is also emerging evidence that GLP-1 receptor agonists have direct protective effects on endothelial cells, promoting vascular repair and preventing abnormal vessel proliferation [13, 14].

A 2020 meta-analysis by Bethel et al. concluded that HbA1c reduction was significantly associated with increased retinopathy risk in patients using GLP-1 receptor agonists. The magnitude of HbA1c reduction was shown to correlate with retinopathy risk [15]. This meta-analysis was not specifically concerned with semaglutide itself, but rather the broader drug class. The long-term outcomes, in regard to retinopathy progression or potential regression, are also unknown.

This trend was further affirmed in the diabetes control and complications trial that found an association between better blood glucose control and the risk of early worsening of diabetic retinopathy [16].

The effects of semaglutide on diabetic retinopathy will be further elucidated with the FOCUS trial. This is the first dedicated ophthalmic study of semaglutide and the eye. This trial is currently in acitve recruitment and is estimated to be complete by 2027. The randomised control trial will examine the effects of semaglutide in diabetic eye disease when compared with placebo. The study will last for 5 years [17]. This is a significantly longer time than that of previous, similar data samples.

## 4. Conclusion

In the interim, it is hoped that more observational evidence will be published demonstrating the positive benefits of semaglutide in the treatment of diabetic retinopathy, as well as the already known benefit of improved glycaemic control.

Unfortunately, in Australia, accessing semaglutide has become challenging for diabetic patients [18]. The demand has increased given the weight loss effects documented of semaglutide in nondiabetic populations [7]. Currently, ophthalmologists are encouraged to regularly review diabetic patients' medications and closely monitor for retinopathy if commenced on a GLP-1 agonist [19].

There may be an independent role for semaglutide in the management of PDR in a similar fashion that fenofibrate is used to reduce the progression of NPDR [20]. The effect noted from this medication is optimistic, and the outcomes of further research are eagerly anticipated. To our knowledge, this is the first report of semaglutide inducing the resolution of PDR.

## Figures and Tables

**Table 1:** HbA1c results around the time of the case report.

**Blood sample collection date**	**HbA1c (%)**
12/11/21	9.0
24/01/22	7.6
5/5/22	9.0
1/8/22—1^st^ reading after semaglutide commencement	9.0

## Data Availability

All data used are included in the manuscripts.
